# The relationship between social media addiction, fear of missing out and family functioning: a structural equation mediation model

**DOI:** 10.1186/s40359-023-01409-7

**Published:** 2023-11-08

**Authors:** Eleonora Topino, Alessio Gori, María Verónica Jimeno, Beatriz Ortega, Marco Cacioppo

**Affiliations:** 1 Department of Human Sciences, LUMSA University of Rome, Via della Traspontina 21, Rome, 00193 Italy; 2 Department of Health Sciences, University of Florence, Via di San Salvi 12, Pad. 26, Florence, 50135 Italy; 3 Department of Psychology, School of Medicine, University of Castilla-La Mancha, Albacete, 02071 Spain; 4 Criminology Research Centre, University of Castilla-La Mancha, Benjamín Palencia Building Campus Universitario s/n, Albacete, 02071 Spain

**Keywords:** Social Media Addiction; problematic social media use, Fear of Missing Out; FoMO, Family Functioning, Mediation Model, SEM; structural equation modeling

## Abstract

**Background:**

The use of social media became a daily activity for many individuals, with recreational, informational, and social purposes, to name a few. However, for some subjects, the use of these platforms may become problematic and generate functioning impairments in many life areas. Given this, the present research aimed at investigating the factor that may contribute to Social Media Addiction, by focusing on Fear of Missing Out and Family Functioning Patterns.

**Methods:**

A sample of 303 social media users (*M*_*age*_ = 35.29; *SD* = 14.87; 65% females, 35% males) completed a survey including the Bergen Social Media Addiction Scale, Family Adaptability and Cohesion Evaluation Scales-IV, and Fear of Missing Out Scale. Data were analysed by implementing Pearson correlation and testing a mediation with the Structural Equation Model approach.

**Results:**

Cohesion, flexibility, and disengagement family functioning patterns were significantly associated with the levels of social media addiction. These dimensions were inserted in the structural equation model, where the full mediation of fear of missing out in their relationship with social media addiction was shown.

**Conclusions:**

The data showed the protective effect of flexible and cohesive family functioning patterns, as well as the role of disengagement and, sequentially, fear of missing out as risk factors. These findings may provide useful indications to elaborate tailored and effective therapeutic and preventive activity.

## Background

The rapid development of information and communication technologies has led to the inclusion of new tools in people's daily lives, which can therefore interact, have fun, learn, and perform numerous specific activities online [1–4]. In this context, social media is a very popular instrument that provides online environments for building and sustaining friendships, exchanging information and/or thoughts, images and/or videos, even in real-time [5]. Although social media can offer numerous benefits and support processes such as the exploration of gender and identity, self-expression, and socialization [6], a growing body of evidence points out that some at-risk individuals can develop an addiction [7, 8]. Social Media Addiction (SMA) could be defined as a dysfunctional and dysregulated form of social media use that hesitates in an irrepressible motivation to access these platforms and in the perpetuation of this behaviour despite the negative consequences in different areas of life, compromising well-being, social and interpersonal relationships, work/school activities [9]. Furthermore, this condition also exhibits the characteristics identified by Griffiths [10] as hallmarks of behavioural addictions: mood modification, salience, tolerance, withdrawal symptoms, conflict, and relapse [10–12]. Furthermore, SMA is associated with both physical and psychological negative consequences, such as sleep disturbances [13], somatic symptoms [14], depression [15], and anxiety [16]. Given its clinical relevance, a growing body of research is addressing the understanding of SMA and the antecedents of this condition (see Lee et al. [17] for a review), in order to facilitate early diagnosis, prevention, as well as risk factors for aims in the therapeutic activity. In this line, the present research aimed at investigating the factor that may contribute to SMA, by placing a specific focus on Fear of Missing Out and Family Functioning Patterns, with the theoretical orientation of the Self-Determination Theory [18, 19].

### The theoretical framework: the self-determination theory

The Self-Determination Theory [18, 19] is a motivational theory based on the relationship perspective. More specifically, according to this view, individual behaviours are motivated by three socio-psychological needs: 1) autonomy, i.e., the need to perceive psychological freedom in one’s actions; 2) competence, i.e., the need to perceive a sense of effectiveness in ones’ actions to realize and obtain desired outcomes; 3) relatedness, i.e., the need to feel connected with other people and cared for. These basic needs are seen as universal and have a central role in the well-being and health of individuals: their satisfaction facilitates flourishing and adaptation [20], while their denial may be the source of compensatory actions aimed at behavioural regulation [21] and this may increase vulnerability to psychopathology [22]. This perspective guided some research about problematic internet use [23, 24], also with a detailed focus on SMA (see Sun & Zhang [25] for a review), suggesting the possibility that a low level of satisfaction with basic needs in the closest environment, such as the family context [24], may lead individuals to turn to external sources to obtain the desired gratifications, favouring a compensatory use of social media and increasing the risk of addictive behaviour [26].

### Family Functioning and Social Media Addiction (SMA)

Family functioning refers to the overall quality of family life [27], and plays a key role in the well-being and mental health of individuals [28, 29]. Focusing on the field of addictions, lower family functioning was found to be associated with alcohol and substance abuse [30, 31], gambling disorder [32], as well as with problematic technology use, such as internet addiction [33], problematic online gaming [34], and SMA [35]. Furthermore, recent empirical evidence has deepened the study of the relationship between family functioning and technological addictions through the application of the Circumplex Model of Marital and Family Systems by Olson and colleagues [27], allowing for the exploration of the good (or balanced) family functioning dimensions considering the levels of cohesion and flexibility, and for the investigation of the poor (or unbalanced) ones analysing the patterns of disengagement, enmeshment, rigidity, and chaos. By applicating Olson's conceptualization [27, 36], previous research provided interesting insights into problematic online gambling [37], problematic smartphone use [38], and compulsive online shopping [39], showing that the different family functioning patterns may present associations with other variables and intervene as risk (the balanced ones) or protective (the unbalanced ones) factors differently based on the specific addiction. In light of this and given evidence supporting the association between family factors and problematic social media use [40], the use of the Circumplex Model of Marital and Family Systems in the analysis of the relationship between family functioning and SMA could be useful to provide more detailed information and further orient the clinical practice in this field.

### Family Functioning and Fear of Missing Out (FoMO)

A large and temporally extended line of research has highlighted that contextual factors may have influences on psychosocial functioning [41, 42]. Consistently, a positive family environment was positively associated with life satisfaction [43], romantic relationship satisfaction [44], and secure peer attachment [45]. On the other hand, previous evidence has shown that the perception of poor family functioning and more family conflicts were significantly associated with lower psychosocial adaptation [46], higher perception of loneliness [47, 48], and a stronger Fear of Missing Out (FoMO) [49]. Fear of Missing Out (FoMO) refers to an experience of constant and pervasive fear and worry of being excluded from gratifying experiences experienced by others [50]. Previous research discussed this phenomenon through the lens of the Self-Determination Theory [18, 19], conceptualizing FoMO as a self-regulator that emerges from deficiencies in meeting psychological needs [50]. Therefore, this perspective suggests the possibility that individuals with lower levels of satisfaction with basic needs in the family context may feel emotionally rejected by this environment, and search for compensation in the external one that could favour FoMO [51].

### Fear of Missing Out (FoMO) and Social Media Addiction (SMA)

Fear of Missing Out (FoMO) is a factor attracting great scientific interest concerning its association with problematic social media use (see Tandon et al. [52] 2021 for a review). High levels of FoMO have a negative impact on individuals' lives, as suggested by previous evidence showing significant relationships between FoMO and depression, anxiety, fear of negative evaluation [53], negative mood, low self-esteem, sense of inadequacy, and lower levels of life satisfaction [50, 54]. Furthermore, in light of the fear of exclusion inherent in this condition, it is not surprising that FoMO has been associated with excessive use of digital communication tools to the point of developing an addiction [55, 56], thus representing a risk factor for problematic internet use [57], for smartphone addiction [58], as well as for SMA [59, 60]. More specifically, the conceptual perspective of the self-determination theory [18, 19] offers a vision of FoMO not as an SMA trigger per se, but as an element of friction that stimulates the search in social media for the fulfilment of some crucial needs that have not been satisfied in other contexts [50], such as the family one.

### The present research

Although the application of Olson's conceptualization [27, 36] proved to be useful and effective in investigating some online problematic behaviours [37, 39], at the time of writing there is still no research exploring the relationships between family functioning, FoMO, and SMA in a single model where the descriptions of good or poor family functioning in line with Circumplex Model of Marital and Family Systems [27, 36]. Furthermore, even if SMA also affects populations over different ages [61, 62], studies including these factors mainly focused on adolescents [63, 64] and evidence on adults considering these variables is still scarce. Given the aforementioned theoretical and empirical framework, the present research aimed to fill this gap by investigating the relationships between factors that may influence the levels of SMA among adult social media users, considering the role of FoMO and family functioning conceptualized according to the Circumplex Model of Marital and Family Systems [27]. First, the relationship between cohesion (balanced family functioning), flexibility (balanced family functioning), disengagement (unbalanced family functioning), enmeshment (unbalanced family functioning), rigidity (unbalanced family functioning), chaos (unbalanced family functioning) and SMA was explored.

Then, considering only the family functioning patterns showing a significant relationship with SMA, a Structural Equation Model was elaborated assuming that FoMO would be significant a mediator in the relationships between the involved family functioning patterns and SMA. More specifically, it was hypothesized that:

- H_1_) The balanced family functioning patterns involved would be significantly and negatively associated with SMA;
- H_2_) The unbalanced family functioning patterns involved would be significantly and positively associated with SMA;
- H_3_) The balanced family functioning patterns involved would be significantly and negatively associated with FoMO;
- H_4_) The unbalanced family functioning patterns involved would be significantly and positively associated with FoMO;
- H_5_) FoMO would be significantly and positively associated with SMA.

## Method

### Participants and procedure

A sample of 303 participants who regularly use social media was involved in this research (see Table 1). Their mean age was 35.29 (*SD* = 14.87; age range = 18 – 81 years) and were predominantly women (65%). Most of them declared to be single (55%), have a high school diploma (40%) and work as employees (34%). They were recruited through snowball sampling starting from the researchers’ social media, and completed the survey online, through the Google Forms platform. The inclusion criteria were: 1) Declare to use social media daily; 2) Declare to have a good command of the Italian language. On the other hand, those who were not at least 18 years old were excluded. Before starting, each participant was informed about the general aim of the research and provided informed consent electronically. The respondents were also told the data were analysed anonymously and in aggregate form. All the procedures performed in the study have been approved by the Ethics Committee for Scientific Research (CERS; study number 003/D178) of the LUMSA University of Rome, Rome, Italy.

**Table 1:** Demographic characteristics of the sample (*N* = 303)

Characteristics		M ± SD	N (*%*)
Age		35.29 ± 14.865	
Sex			
	*Males*		106 (35.0%)
	*Females*		197 (65.0%)
Marital Status			
	*Single*		166 (54.8%)
	*Married*		60 (19.8%)
	*Cohabiting*		62 (20.5%)
	*Separated*		2 (0.7%)
	*Divorced*		10 (3.3%)
	*Widowed*		3 (1.0%)
Education			
	*Middle School diploma*		12 (8.3%)
	*High School diploma*		120 (39.6%)
	*University degree*		70 (23.1%)
	*Master’s degree*		76 (25.1%)
	*Post-lauream specialization*		25 (8.3%)
Occupation			
	*Student*		60 (19.8%)
	*Working student*		46 (15.2%)
	*Artisan*		5 (1.7%)
	*Homemaker*		7 (2.3%)
	*Trader*		2 (0.7%)
	*Employee*		103 (34.0%)
	*Manager*		2 (0.7%)
	*Entrepreneur*		8 (2.6%)
	*Freelance*		32 (10.6%)
	*Retired*		24 (7.9%)
	*Unemployed*		14 (4.6%)

### Measures

#### Bergen Social Media Addiction Scale (BSMAS)

The *Bergen Social Media Addiction Scale* (BSMAS; Andreassen et al. [65]; Italian version: Monacis et al. [66]) is a 6-item self-report scale used to assess the levels of problematic social media use, in line with the components model of behavioural addiction [10]. Items are rated on a five-point Likert scale, from 1 (“*very rarely*”) to 5 (“*very often*”). Higher scores indicate higher levels of problematic social media use. The total score of the Italian version was used in this research [66] and showed acceptable internal consistency in the present sample (*α* = 0.74).

### Family Adaptability and Cohesion Evaluation Scales-IV (FACES IV)

The *Family Adaptability and Cohesion Evaluation Scales-IV* (FACES IV; Olson [36]; Italian version: Baiocco et al. [67]) is a 42-item self-report scale used to assess the level of some family functioning dimensions, based on the Circumplex Model of Marital and Family Systems [27, 36]. Items are rated on a five-point Likert scale, from 1 (“*Strongly Disagree*”) to 5 (“*Strongly Agree*”), and may be grouped into six subscales: the “Cohesion” and “Flexibility ones indicating features of the balanced functioning, and the “Enmeshed”, “Disengaged”, “Chaotic”, and “Rigid” ones indicating features of the unbalanced functioning. The Italian version was used in this research [67], and all six scales showed satisfactory internal consistency in the present sample (Cohesion, *α* = 0.83; Flexibility, *α* = 0.75; Enmeshed, *α* = 0.71; Disengaged, *α* = 0.68; Chaotic, *α* = 0.62; Rigid *α* = 0.0.72).

### Fear of Missing Out Scale (FoMOs)

The *Fear of Missing Out Scale* (FoMO; Przybylski et al. [50]; Italian version: Casale & Fioravanti, [68]) is a 10-item self-report scale used to assess the levels of apprehension that others might be having rewarding experiences in which the respondent is not participating. Items are rated on a five-point Likert scale, from 1 (“*Not at all true of me*”) to 5 (“*Extremely true of me*”), and may be grouped into two subscales: Fear and Control. Higher scores indicate higher levels of fear of missing out. The Italian version was used in this research [68] and showed good internal consistency in the present sample (Total score, *α* = 0.82; Fear, *α* = 0.82; Control, *α* = 0.72).

### Data analysis

The SPSS (v. 21.0; IBM, New York, USA) and AMOS (v. 24.0; IBM, New York, USA) software for Windows were used to perform the analyzes. A *p* < 0.05 value was considered as the threshold of statistical significance. Pearson correlation analysis was implemented to evaluate the associations between the variables. Based on this investigation, a Structural Equation Model (SEM) [69] was elaborated to analyze the relationship between family functioning and social media addiction with the mediation of fear of missing out, by including in the model only the subdimensions of family functioning that showed a significant correlation with social media addiction. The statistical goodness of fit of the model was assessed based on a range of indices: the Chi-square (*χ*^*2*^) of the model, indicating a good fit when *p* > 0.05 [70]; the Goodness of Fit (GFI), Normed-Fit Index (NFI), Tucker Lewis index (TLI) and Comparative Fit Index (CFI) indicating a good fit when the values are above 0.95 [71, 72]; the Root Mean Square Error Of Approximation (RMSEA) and Standardized Root Mean Square Residual (SRMR), indicating a reasonable fit when the values are below 0.08 [70, 73]. Then, the statistical stability of the model was assessed by exploring significance of the total, direct and indirect paths also performing the bootstrap technique (5000 bootstrapped samples with 95% Confidence Interval) [74], confirming the significance of the effects when the 95% bias-corrected bootstrap confidence intervals (from Lower Limit Confidence Interval [Boot LLCI] to Upper Limit Confidence Interval [Boot ULCI]) did not contain zero.

## Results

As shown in Table 2, Social media addiction showed significant correlations with cohesive (*r* = -0.472, *p* < 0.01), flexible (*r* = -0.462, *p* < 0.01), and disengaged (*r* = 0.332, *p* < 0.01) family functioning subdimensions. Furthermore, Social media addiction was significantly and positively associated with FoMO, both considering the total score (*r* = 0.434, *p* < 0.01) and the subscales (Fear, *r* = 0.414, *p* < 0.01; Control, *r* = 0.357, *p* < 0.01).

**Table 2:** Correlation matrix

	1	2	3	4	5	6	7	8	9	10
1. Social Media Addiction	1	0.434**	0.414**	0.357**	-0.472**	-0.462**	0.332**	0.083	0.031	0.073
2. Fear of Missing Out (Total score)		1	0.841**	0.908**	-0.474**	-0.451**	0.413**	0.174**	0.128*	0.110
3. Fear (Fear of Missing Out)			1	0.536**	-0.453**	-0.417**	0.322**	0.156**	0.081	0.060
4. Control (Fear of Missing Out)				1	-0.388**	-0.381**	0.394**	0.151**	0.137*	0.125*
5. Cohesive Family Functioning					1	0.789**	-0.486**	-0.085	-0.060	-0.039
6. Flexible Family Functioning						1	-0.375**	-0.076	0.078	-0.094
7. Disengaged Family Functioning							1	0.280**	0.330**	0.385**
8. Enmeshed Family Functioning								1	0.539**	0.283**
9. Rigid Family Functioning									1	0.043
10. Chaotic Family Functioning										1

Bold values indicate significant *p*-values. **. Correlation is significant at the 0.01 level (2-tailed). *. Correlation is significant at the 0.05 level (2-tailed)

**Fig. 1:**
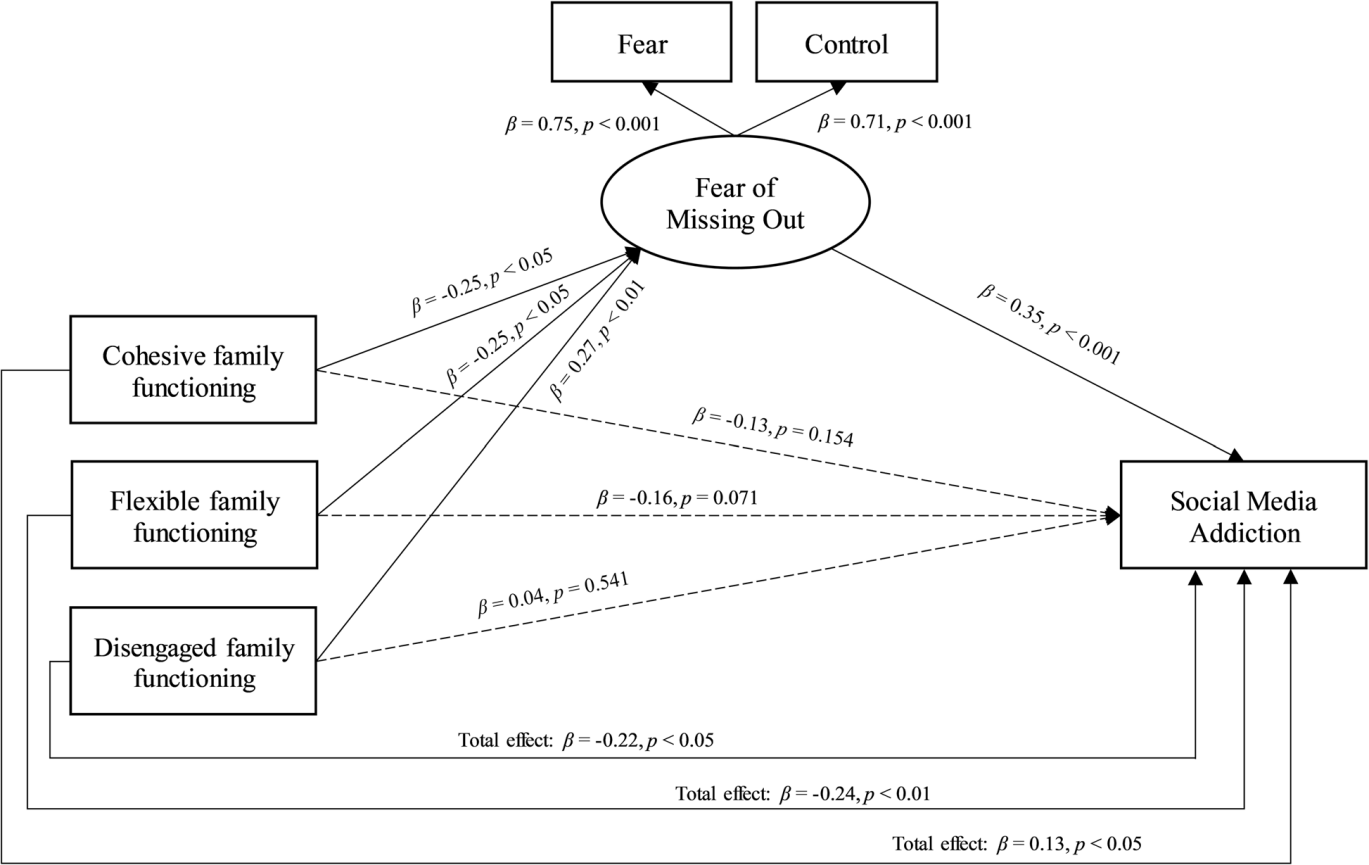
The mediation of FoMO in the relationship between Cohesive, Flexible, and Disengaged Family Functioning patterns and SMA

On this basis, cohesive, flexible, and disengaged family functioning were the only patterns included in the SEM, where the mediation of fear of missing out in their relationship with social media addiction was explored. The emerging mediation model showed an excellent fit to the data: *χ*^2^ (3) = 6.760 (*p* = 0.080), GFI = 0.993, NFI = 0.990; TLI = 0.972, CFI = 0.994, RMSEA = 0.064, SRMR = 0.017 (see Fig. 1).

Specifically, significant total effects were shown in the relationships between cohesive (*β* = 0.13, *p* < 0.05, H_1_), flexible (*β* = 0.24, *p* < 0.01, H_2_), and disengaged (*β* = -0.22, *p* < 0.05, H_2_) family functioning patterns with social media addiction. Furthermore, the involved family patterns were significantly associated with FoMO (cohesive, *β* = -0.25, *p* < 0.05, H_3_; flexible, *β* = -0.25, *p* < 0.05, H_3_; disengaged, *β* = 0.27, *p* < 0.01, H_4_). In turn, FoMO was significantly and positively related to social media addiction (*β* = 0.35, *p* < 0.001, H_5_), such that, when included in the model, FoMO totally mediated the effect of cohesive, flexible, and disengaged family functioning patterns on social media addiction (see Table 3), determining non-significant direct effects (*β* = -0.13, *p* = 0.154; *β* = -0.16, *p* = 0.071; *β* = 0.04, *p* = 0.541, respectively).

**Table 3:** Coefficients of the structural equation mediation model

	Estimate	SE	*p*	BootLLCI	BootULCI
*Total effects*					
Cohesive family functioning → Social media addiction	-0.150	0.059	< 0.05	-0.264	-0.033
Flexible family functioning → Social media addiction	-0.183	0.062	< 0.01	-0.308	-0.063
Disengaged family functioning → Social media addiction	0.110	0.049	< 0.05	0.014	0.209
*Direct effects*					
Cohesive family functioning → Social media addiction	-0.089	0.062	0.153	-0.210	0.036
Flexible family functioning → Social media addiction	-0.118	0.065	0.071	-0.247	0.009
Disengaged family functioning → Social media addiction	0.035	0.056	0.542	-0.077	0.036
*Indirect effects*					
Cohesive family functioning → Social media addiction	-0.061	0.032	< 0.01	-0.144	-0.013
Flexible family functioning → Social media addiction	-0.066	0.033	< 0.01	-0.150	-0.017
Disengaged family functioning → Social media addiction	0.075	0.033	< 0.001	0.025	0.158

Finally, the bias-corrected bootstrap procedure (5000 bootstrapped samples) confirmed the statistical stability of the full structural equation mediation model (see Table 3).

## Discussion

Although it has not yet been incorporated into the mainstream nosology systems used by mental health professionals [75], SMA has attracted considerable attention from the academic community, given the exponential growth in the use of social media in daily life and the significance and pervasiveness of the negative consequences for those who develop a problematic use of these platforms [76]. Therefore, the present research aimed at investigating the factor that may contribute to SMA, by specifically focusing on Family Functioning and Fear of Missing Out, according to the theoretical guide of the Self-Determination Theory [18, 19]

Concerning Family Functioning Patterns, both the cohesion and flexibility dimensions were significantly and negatively associated with SMA, further supporting the relationship between balanced family functioning and mental health [28, 77]. Regarding cohesion, defined as the positive emotional bond and the feeling of closeness that family members have towards each other [36], the data obtained are consistent with previous studies showing its protective role on other technological addictions, such as gambling problematic online gambling [37] and compulsive online shopping [39]. Furthermore, a previous study by Paolini and colleagues [32] involving a clinical sample of pathological gamblers highlighted that they showed medium–low scores both in cohesion and flexibility, a result which supports, in line with the findings of the present research, also the inverse relationship between addiction and flexible family functioning, which could be defined as the quality and clarity of leadership and organization, roles, relational rules and negotiations [36]. Referring to the Unbalanced Family Functioning Patterns, the dimension of disengagement was found to be significantly and positively associated with SMA, in line with other types of internet addiction [78]. Disengaged families are characterized by limited commitment and connection to other family members [79], high levels of autonomy, little warmth, affection and reciprocal interconnection [80], and these results, therefore, suggest the possibility that social media could become a means to search for compensation for the emotional support that is lacking in the family context [81].

Such findings were further deepened and enriched by the implementation of the structural equation model, which showed that the relationships between the involved Family Functioning Patterns (cohesion, flexibility, and disengagement) and SMA totally were mediated by FoMO, supporting all the hypotheses (H_1_ to H_5_). This echoes and detailed previous results, highlighting a significant mediation of FoMO in the negative associations between family communication and problematic internet use [82]. According to the Self-Determination Theory [18, 19], when the family context is balanced (characterized by cohesion and flexibility), this seems to represent a protective factor that offers the subject the necessary resources to determine himself without leading to pathological ways of using social media [35]. Conversely, when family functioning is dysfunctional with high levels of disengagement, it does not meet the individual's basic needs, and this could result in a dysfunctional attempt to compensate for this deficiency online, seeking in social media the warmth that does not receive offline. In this framework, therefore, FoMO acquires the role of mediator, originating from a deficit in psychological needs and driving towards the search for self-determination in online platforms to such a level as to develop an addiction [50, 51]. Consistently with this, in fact, a recent meta-analysis showed that the relationship between FoMO and SMA was found to be stronger than the relationship between FoMO and non-problematic use of social networks [53].

The present study has some limitations that need to be considered. First, the cross-sectional design did not allow for establishing certain inferences regarding the direction of relationships. Although this research is in line with the theoretical perspective of the Self-Determination Theory [18, 19] and the explored relationships were supported by previous evidence, family conflicts and problems may also be consequences of psychosocial deterioration due to addiction [83]. Longitudinal research is needed in future research to delineate causal relationships more rigorously. Furthermore, most of the participants declared themselves to be single and to be students or working students, and a significant percentage were female. This could influence the type, timing, and impact of social media use, requiring caution in generalizability of results to other categories. Consistently, no information was collected regarding these data and any differences in this regard were not explored. An interesting challenge for future research could be the investigation of these aspects, also verifying the replicability of the results in samples having different features. In this line, participants may have completed the FACES-IV [36, 67] considering the family of origin or stepfamily, since no indications in this regard were provided in the instructions. Indeed, the focus of this study was the exploration of the perception of family functioning, regardless of which family was more salient. However, future research could expand these results, by acquiring information on this aspect as well and investigating any differences. In addition, the disengaged and chaotic family functioning patterns assessed through the FACES-IV showed Cronbach’s alpha values < 0.70 in the present sample. Although some authors support the possibility of considering values of 0.60 acceptable [84], future research may confirm the results of this study by using measures with higher internal consistency. Moreover, the collected data were self-report, and this exposes the risk of biases. A multi-method approach (e.g., by integrating experimental methods) could be used in future research to overcome this issue. Despite these limitations, the present study is part of a research line aimed at identifying the factors that can be associated with the vulnerability, maintenance and severity of addictions [85–87], both those related to substance abuse and the behavioural ones [88–93]. Therefore, by placing a specific focus on SMA, the results of this research can further stimulate future research related to technological addiction and provide useful information for developing tailored preventive and clinical interventions.

## Conclusions

The continuous growth of the popularity of new technologies and their constant development has stimulated research on the potential benefits of their use [3], but also and above all on the exploration of problematic ways of using these tools [94–96]. Given this context, the present research focused on the relationship between family functioning patterns, FoMO, and SMA. Results highlighted that FoMO significantly mediated the relationship between cohesive, flexible, and disengaged family functionings and SMA. Such data on one hand suggest the protective effect of balanced family functioning, and on the other hand, identified disengagement and, sequentially, FoMO, as significant risk factors. These findings may provide useful indications to elaborate tailored and effective clinical practice and preventive activity, by highlighting both protective and risk factors towards which to direct interventions.

## Data Availability

The datasets used and/or analysed during the current study are available from the corresponding author on reasonable request.
